# State-level metabolic comorbidity prevalence and control among adults age 50-plus with diabetes: estimates from electronic health records and survey data in five states

**DOI:** 10.1186/s12963-022-00298-z

**Published:** 2022-12-02

**Authors:** Russell Mardon, Joanne Campione, Jennifer Nooney, Lori Merrill, Maurice Johnson, David Marker, Frank Jenkins, Sharon Saydah, Deborah Rolka, Xuanping Zhang, Sundar Shrestha, Edward Gregg

**Affiliations:** 1 Westat, 1600 Research Blvd, Rockville, MD 20850 USA; 2 US Centers for Disease Control and Prevention, 1600 Clifton Rd., Atlanta, GA 30329 USA

**Keywords:** Diabetes mellitus, Electronic health records, Epidemiologic methods, High cholesterol, Hypertension, Health and Retirement Study, National Health and Nutritional Examination Survey, Population surveillance

## Abstract

**Background:**

Although treatment and control of diabetes can prevent complications and reduce morbidity, few data sources exist at the state level for surveillance of diabetes comorbidities and control. Surveys and electronic health records (EHRs) offer different strengths and weaknesses for surveillance of diabetes and major metabolic comorbidities. Data from self-report surveys suffer from cognitive and recall biases, and generally cannot be used for surveillance of undiagnosed cases. EHR data are becoming more readily available, but pose particular challenges for population estimation since patients are not randomly selected, not everyone has the relevant biomarker measurements, and those included tend to cluster geographically.

**Methods:**

We analyzed data from the National Health and Nutritional Examination Survey, the Health and Retirement Study, and EHR data from the DARTNet Institute to create state-level adjusted estimates of the prevalence and control of diabetes, and the prevalence and control of hypertension and high cholesterol in the diabetes population, age 50 and over for five states: Alabama, California, Florida, Louisiana, and Massachusetts.

**Results:**

The estimates from the two surveys generally aligned well. The EHR data were consistent with the surveys for many measures, but yielded consistently lower estimates of undiagnosed diabetes prevalence, and identified somewhat fewer comorbidities in most states.

**Conclusions:**

Despite these limitations, EHRs may be a promising source for diabetes surveillance and assessment of control as the datasets are large and created during the routine delivery of health care.

*Trial Registration*: Not applicable.

**Supplementary Information:**

The online version contains supplementary material available at 10.1186/s12963-022-00298-z.

## Background

Control of Hemoglobin **A**1c, **B**lood Pressure, and **C**holesterol (ABCs) is essential for preventing micro- and macro-vascular diabetes-related complications. The lack of state-level estimates of the prevalence and control of diabetes and major metabolic comorbidities, such as hypertension and high cholesterol, limits the ability of public health agencies to monitor diabetes prevention and management at the state level [1]. Data from electronic health records (EHRs), along with novel uses of survey data, may be able to fill gaps in the nation’s diabetes surveillance system [2–4]. However, more work is needed to validate estimates from these non-traditional methods and data sources. Data from EHRs are challenging to analyze for population-based studies because they are generated from the routine delivery of clinical care. Therefore, they cover partial, sometimes non-representative subpopulations, do not always include the same measurements on all individuals, and include limited variables for case finding and adjustment [5].

We could find no previous studies that examined metabolic comorbidities and control within the diabetes population at the state level. Researchers in New York City validated EHR-based estimates in the general adult population relative to population-based survey and chart review data. The EHR-based estimates performed well for diabetes and hypertension, although the authors recommended using EHR data for high cholesterol with caution [6, 7]. Several studies examined EHR data from participating health systems to describe metabolic risk factors among diabetes patients, but they do not address the representativeness of this group in the broader population [8, 9]. Other analysts looked at control of the ABCs within the diabetes population at the national level, but did not examine state-level data [10–13]. In follow-up work, the methods developed in this paper were applied to NHANES data to create estimates of ABC control for each state in the USA. [14]

The purpose of this analysis is to compare EHR-based and survey-based estimates of the prevalence of comorbid hypertension and high cholesterol, and ABC control, in the diabetes population at the state level.

## Methods

We compared EHR data from the DARTNet Institute, a collaboration of practice-based research networks, with estimates derived from two surveys: (1) the National Health and Nutrition Examination Survey (NHANES), used to create synthetic state-level estimates by adjusting national estimates to State demographic population counts, and (2) the Health and Retirement Study (HRS), used to create synthetic state-level estimates based on within-state or neighboring-state data. We analyzed five states representing diverse geography and populations (Alabama, California, Florida, Louisiana, and Massachusetts). We focused on those age 50 and over who had an office visit in the past year because they are included in each dataset.

### Data sources

#### DARTNet EHR data 2012–2013

The DARTNet Institute is a national collaboration of practice-based research networks that has built a collection of data from electronic health records, claims, and patient-reported outcomes. Eight participating networks contributed diagnosis and prescription information, patient vitals, and ABC measurements. We used ABC results from 2012 and 2013 for the control analysis to better match the survey timeframes, and we used diagnosis, biomarker, and prescription information from 2010 to 2013 for case finding. The EHR data did not include fasting plasma glucose (FPG) values.

#### NHANES 2011–2012

NHANES is a non-institutionalized population-based survey that includes ABC measurements on all participants, as well as variables useful for adjustment such as age, sex, race/ethnicity, insurance type, education, marital status, income, healthcare visit in the past 12 months, and general health status. We used the public-use file, which does not include a state residence variable. NHANES is not designed for state-level estimation, and the dataset may not contain respondents from each state. By design, the NHANES data included A1c values for the whole sample and FPG values for a random half of the sample. We used a previously developed imputation model for diabetes status that performed well and accounted for non-measured FPG values [15].

#### HRS 2010–2012

HRS is a longitudinal panel study that surveys a representative sample of approximately 20,000 non-institutionalized Americans over the age of 50 every 2 years. HRS is not designed for direct state-level estimation. In addition to self-reported data on diabetes and other chronic diseases, HRS includes ABC measurements on all participants, as well as many variables for adjustment. HRS does not include FPG values, but it had similar covariates to NHANES so we applied the diabetes status imputation model developed using NHANES data to account for non-measured FPG [15].

### Dataset preparation

Depending upon the data source, the information available in addition to the ABC values includes survey responses, diagnosis codes, prescriptions written, and other variables useful for modeling and adjustment. Prior to analysis, we harmonized variables across datasets so similar concepts were coded as consistently as possible.

For the analysis of the NHANES data, we created synthetic state-level estimates. We adjusted NHANES national weights to reflect the demographic characteristics of each target state by raking and propensity modeling [16–21]. This resulted in person-level adjusted weights that match the state-level distributions of selected demographic variables from the American Community Survey, and health status from the Behavioral Risk Factor Surveillance System. For HRS, we were able to create estimates for the two largest states (California and Florida) based on respondents from those states alone. For the other three states, we created synthetic state estimates using respondents in the census division in a manner similar to the NHANES adjustment. Additional details of the adjustments for NHANES and HRS are provided elsewhere [15].

The EHR data were originally in six datasets containing multiple records per person for visits, diagnoses, laboratory tests, prescription drugs ordered, providers, and demographic characteristics. We merged records from these files to create an analytic file with a single comprehensive record for each individual that included variables representing disease status, ABC results, prescription history, and age and sex. The EHR data preparation process is illustrated in Additional file 1: Online Appendix Figure A1.

**Fig. 1:**
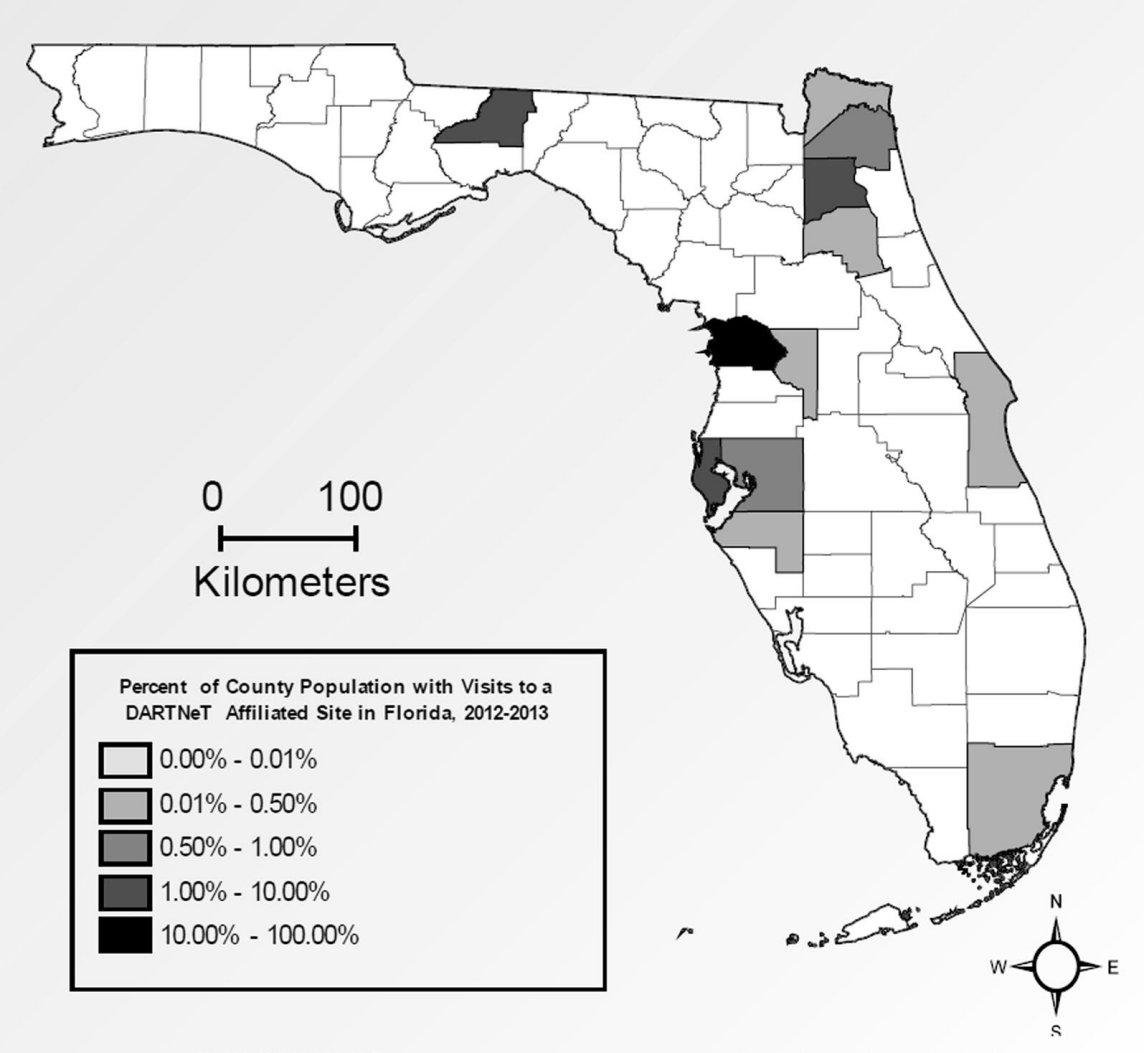
Geographic distribution of patients in the EHR dataset, Florida 2012–2013

The EHR dataset includes clinic ZIP code, allowing us to assess the geographic distribution of clinic visits. In each state, visits were highly clustered by county. For example, Fig. 1 illustrates the geographic clustering in Florida. There are clusters in several urban areas, and relatively few visits in other areas. We designed a two-step weighting strategy to improve the geographic representation within each state. First, cases within each county were weighted to county-level American Community Survey totals of age and sex cross-tabulations. This ensures that each county in the dataset receives a total weight proportional to its total population. Second, the weighted totals of age by sex groups for the counties represented in the data were raked to the corresponding state totals for those with an office visit in the past year.

### Significance testing and precision of estimates

We quantified the precision of the NHANES and HRS estimates with confidence intervals (CIs) calculated using standard errors that take into account the sample design, and used a t test to calculate whether differences in estimates across the datasets were statistically significant. For the EHR data, we calculated CIs and t tests that incorporate the geographic weights described above.

### Case and disease control definitions

We developed case definitions for diabetes, hypertension, and high cholesterol, and for control of the ABCs, that could be applied across the data sources. Additional details can be found in Additional file 2: Online Appendix Tables A1, A2, and A3.

**Table 1:** Diabetes prevalence by data source and state among adults age 50-plus with an office visit in the past year

	NHANES, adjusted estimates 2011–2012		HRS, adjusted estimates 2010–2012		EHR, adjusted estimates 2012–2013	
	% of population	95% CI	% of population	95% CI	% of population	95% CI
*Alabama*						
Diagnosed diabetes	22.6	20.6, 24.6	27.0	21.7, 32.2	18.0↓↓	13.9, 22.2
Undiagnosed diabetes	3.4	2.6, 4.3	4.0	2.2, 5.8	0.2↓↓	0, 4.1
*California*						
Diagnosed diabetes	15.4↓↓	13.0, 17.9	20.5	18.0, 23.0	24.6↑↑	20.5, 28.8
Undiagnosed diabetes	5.2↑	3.7, 6.7	3.4	1.5, 5.3	1.6↓	0, 5.6
*Florida*						
Diagnosed diabetes	17.4↓	15.5, 19.3	24.3↑	18.0, 30.6	19.0	14.9, 23.1
Undiagnosed diabetes	3.7	3.0, 4.5	2.8	1.0, 4,6	0.7↓↓	0, 4.7
*Louisiana*						
Diagnosed diabetes	23.1↑	20.2, 26.0	20.3	16.8, 23.9	18.0↓	14.0, 22.0
Undiagnosed diabetes	3.7↑	2.7, 4.6	3.7	0.0, 7.6	0.3↓	0, 4.2
*Massachusetts*						
Diagnosed diabetes	15.3↓	11.6, 19.0	18.6	15.7, 21.5	20.1↑	16.0, 24.1
Undiagnosed diabetes	3.2↑↑	2.5, 3.9	1.7	0.9, 2.5	1.2	0, 5.1

↑ (↓) indicates significantly above (below) one of the other data sources (*p* < .01); ↑↑ (↓↓) indicates significantly above (below) both of the other data sources (*p* < .01)

*NHANES* National Health and Nutrition Examination Survey, *HRS* Health and Retirement Study, *EHR* electronic health record, *CI* confidence interval. All NHANES estimates are synthetically derived, based on adjusted data

**Table 2:** Hypertension and high cholesterol prevalence in the diabetes population by data source and state among adults age 50-plus with an office visit in the past year

	NHANES, adjusted estimates 2011–2012		HRS, adjusted estimates 2010–2012		EHR, adjusted estimates 2012–2013	
	% of population	95% CI	% of population	95% CI	% of population	95% CI
*Alabama*						
High cholesterol	69.9	63.3,76.6	75.9↑	67.2, 84.6	63.0↓	57.3, 68.7
Hypertension	78.1	72.0, 84.1	87.9 ↑↑	80.6, 95.1	68.5↓↓	62.8, 74.2
Both	56.8	50.6, 63.1	70.2↑↑	59.1, 81.3	45.6↓↓	40.0, 51.2
Neither	9.0	6.1, 11.9	6.4	0.8, 12.1	14.1↑↑	9.0, 19.2
*California*						
High cholesterol	72.8↑	67.0, 78.5	65.1	55.1, 75.1	64.2↓	59.4, 69.0
Hypertension	74.9	68.8, 81.1	82.3	73.4, 91.1	65.5↓↓	60.6, 70.3
Both	56.0	48.6, 63.4	57.3	47.4, 67.2	43.4↓↓	38.6, 48.2
Neither	8.8↓	5.2, 12.3	10.0	2.0, 18.0	13.8↑	9.3, 18.3
*Florida*						
High cholesterol	68.8	63.5, 74.0	73.4	64.8, 82.1	53.2↓↓	48.2, 58.1
Hypertension	77.6	72.5, 82.6	78.5	72.9, 84.1	62.8↓↓	59.8, 65.7
Both	55.7	49.5, 62.0	59.8	53.1, 66.6	31.5↓↓	26.9, 36.2
Neither	9.6	6.0, 13.3	7.9	2.9, 12.9	15.6↑↑	11.0, 20.2
*Louisiana*						
High cholesterol	69.7↑	64.1, 75.3	63.7	54.5, 72.9	58.9↓	54.2, 63.7
Hypertension	80.6	75.2, 86.1	84.1	76.8, 91.5	77.6	73.2, 82.0
Both	58.4	52.0, 64.8	57.5	49.1, 66.0	46.2↓↓	41.4, 50.9
Neither	8.4	5.3, 11.4	9.7	2.7, 16.7	9.6	5.4, 13.8
*Massachusetts*						
High cholesterol	70.8	64.7, 76.9	69.7	61.2, 78.3	82.1↑↑	77.6, 86.5
Hypertension	79.9	74.5, 85.4	81.1	74.6, 87.6	90.9↑↑	86.6, 95.1
Both	59.0	51.9, 66.1	61.0	52.5, 69.4	76.4↑↑	71.8, 80.9
Neither	8.4	5.0, 11.9	10.2	3.8, 16.5	3.4↓↓	0, 7.5

↑ (↓) indicates significantly above (below) one of the other data sources (*p* < .01); ↑↑ (↓↓) indicates significantly above (below) both of the other data sources (*p* < .01)

*NHANES* National Health and Nutrition Examination Survey, *HRS* Health and Retirement Study, *EHR* electronic health record, *CI* confidence interval. All NHANES estimates are synthetically derived, based on adjusted data

**Table 3:** Control of the ABCs in the diagnosed diabetes population by data source and state among adults age 50-plus with an office visit in the past year

	NHANES, adjusted estimates 2011–2012		HRS, adjusted estimates 2010–2012		EHR, adjusted estimates 2012–2013	
	% in Control	95% CI	% in Control	95% CI	% in Control	95% CI
*Alabama*						
A1c	86.1	79.8, 92.3	95.5↑↑	91.0, 100.0	89.3	84.4, 94.2
Blood pressure	71.2	64.1, 78.3	69.6	59.1, 77.2	66.6	61.2, 71.9
Cholesterol	58.2	46.3, 70.1	64.9↑	52.6, 77.2	52.1↓	45.6, 58.6
All three	40.1	28.6, 51.6	45.7	32.5, 59.0	20.6↓↓	14.7, 26.4
*California*						
A1c	86.8	81.1, 92.4	91.1↑	86.3, 95.9	85.4↓	80.8, 89.9
Blood pressure	73.1	65.7, 80.5	56.2↓↓	47.2, 65.3	71.3	66.4, 76.2
Cholesterol	55.3	46.7, 63.8	49.2↓	35.0, 63.4	63.2↑	58.2, 68.3
All three	37.8	30.9, 44.7	22.3↓↓	16.4, 28.2	40.7	35.3, 46.1
*Florida*						
A1c	85.9	80.2, 91.7	92.1	87.8, 96.4	89.6	84.4, 94.8
Blood pressure	70.9	64.5, 77.3	72.4	60.9, 83.9	61.1↓↓	56.1, 66.2
Cholesterol	57.6	48.9, 66.2	47.1	29.7, 64.6	57.7	50.7, 64.6
All three	39.6↓	32.4, 46.8	36.6	18.0, 55.2	48.0↑	43.0, 53.1
*Louisiana*						
A1c	84.6	77.4, 91.8	90.4	82.5, 98.3	87.8	83.0, 92.6
Blood pressure	67.3	63.4, 71.3	72.0	65.8, 78.1	58.7↓↓	53.9, 63.5
Cholesterol	55.3	46.2, 64.5	48.8	36.9, 60.6	59.7	54.0, 65.3
All three	35.8	28.6, 43.0	38.0	27.8, 48.3	35.8	30.2, 41.4
*Massachusetts*						
A1c	84.9↓	77.6, 92.2	93.8↑	89.2, 98.4	89.5	85.1, 93.9
Blood pressure	72.4	65.2, 79.7	64.6	54.6, 74.6	72.9	68.2, 77.6
Cholesterol	58.8	47.5, 70.1	54.7	45.9, 63.5	59.0	54.0, 63.9
All three	42.0	31.8, 52.2	37.6	29.7, 45.4	40.2	35.2, 45.2

↑ (↓) indicates significantly above (below) one of the other data sources (*p* < .01); ↑↑ (↓↓) indicates significantly above (below) both of the other data sources (*p* < .01)

*NHANES* National Health and Nutrition Examination Survey, *HRS* Health and Retirement Study, *EHR* electronic health record, *CI* confidence interval. All NHANES estimates are synthetically derived, based on adjusted data

#### Diabetes

We classified individuals into three categories: diagnosed diabetes, undiagnosed diabetes, or no diabetes. Pregnant women were excluded from all analyses. For NHANES and HRS, diagnosed diabetes was based on self-report of ever being diagnosed by a healthcare provider, excluding gestational diabetes. Patients in the EHR data with at least one ABC result in 2012–2013 were assigned a diabetes status using diagnosis codes and drug prescriptions recorded in 2013 or earlier [22–25]. We used the Healthcare Effectiveness Data and Information Set Comprehensive Diabetes Care pharmacy list as our source for anti-diabetic prescription drugs. Individuals in each dataset who were not diagnosed were classified as undiagnosed cases if they had A1c ≥ 6.5% or FPG ≥ 126 mg/dl in any laboratory result [26].

#### Hypertension

We required at least one hypertension diagnosis code, two or more elevated systolic or diastolic blood pressure readings (systolic ≥ 140 mmHg or diastolic ≥ 90 mmHg), or a prescription for blood pressure medication to identify cases of hypertension [27, 28]. We did not distinguish between diagnosed and undiagnosed hypertension.

#### High cholesterol

We required at least one high cholesterol diagnosis code, a self-report or documentation of drugs to lower cholesterol, or a laboratory result of non-HDL-C ≥ 130 mg/dL to identify cases of high cholesterol. We used non-HDL-C, calculated as total cholesterol minus HDL-C, as the lipid measure since the HRS dataset does not include low-density lipoprotein cholesterol (LDL-C) values, and only 50% of those in the NHANES dataset had an LDL-C value by design [29]. Evidence suggests that non-HDL-C is a good marker of risk in both primary and secondary prevention studies of atherosclerotic cardiovascular disease [30], and the cut point of non-HDL-C ≥ 130 mg/dL to identify high cholesterol has been established in the literature [31]. We did not distinguish between diagnosed and undiagnosed high cholesterol.

#### ABC control

For this study, we used an A1c cut point of < 9% to represent “not-poorly controlled” diabetes (1, [32]. This target is less stringent than the American Diabetes Association’s Standards of Medical Care’s target of A1c < 7.0% for most people with diabetes [26]. Less stringent A1c goals may be appropriate for patients with a history of severe hypoglycemia, the very elderly, extensive comorbid conditions, or long-standing diabetes [33]. Consistent with National Committee for Quality Assurance’s 2016 criteria for the Comprehensive Diabetes Care measure and the ADA’s guidelines, we defined systolic blood pressure of < 140 and diastolic blood pressure < 90 as “adequate control” [22, 34, 35]. While cholesterol control targets for patients with diabetes may be individualized based on personal risk, we used a control target of non-HDL-C < 130 mg/dL.

## Results

As shown in Table 1, the diagnosed diabetes prevalence estimates were higher in the HRS-adjusted data than in the NHANES-adjusted data in four out of five states, significantly so in California and Florida (*p* < 0.01). There were no significant differences between the surveys for undiagnosed diabetes. The EHR prevalence estimates for diagnosed diabetes were more highly variable, being significantly higher than the surveys in some states and significantly lower in others. The EHR-based estimates for undiagnosed diabetes were consistently low across all of the states.

**Fig. 2:**
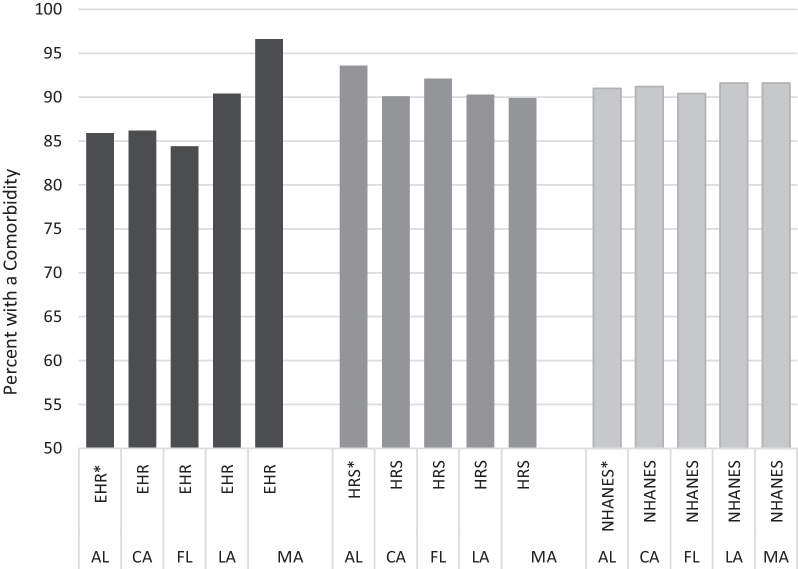
Proportion of the diabetes population with hypertension and/or high cholesterol by data source and state among adults age 50-plus with an office visit in the past year. *NHANES* National Health and Nutrition Examination Survey, *HRS* Health and Retirement Study, *EHR* electronic health record. All NHANES estimates are synthetically derived, based on adjusted data

As shown in Table 2, the prevalence of major metabolic comorbidities within the diabetes population is high. The adjusted NHANES and HRS estimates are not significantly different except in Alabama, where the hypertension and “Both” condition estimates are significantly higher in HRS (*p* < 0.01). In most states, the EHR data show significantly fewer diabetes patients with one or both comorbidities. Massachusetts is a notable exception in that the EHR-based prevalence is significantly higher for both hypertension and high cholesterol. Figure 2 illustrates the variability across states in the prevalence of one or both comorbidities by data source.

Nearly all of the individuals in the NHANES or HRS survey samples had all three ABCs measured. In the EHR data, only 52% of the patients with diagnosed diabetes had A1c measurements, 96% had blood pressure measurements, 46% had cholesterol measurements, and 41% had all three measurements. The EHR-based control percentages shown in Table 3 are based on only those patients who had the relevant test(s). In Alabama, the EHR estimates for control of all three ABCs are lower, significantly so for cholesterol and all three indicators (*p* < 0.01). In addition, the EHR estimates for blood pressure control are significantly lower in Florida and Louisiana. In California, the HRS rates for blood pressure and cholesterol control are significantly low, driving a lower rate for control of all three ABCs. In addition, the A1c control rate is significantly higher in HRS in several states. Otherwise, there is no consistent pattern across the other data sources and states, with most of the differences not significant.

To facilitate comparisons across datasets, we analyzed those aged 50-plus who had contact with the healthcare system in the past year, the subpopulation represented in all three datasets. For completeness, we also looked at pair-wise comparisons of the datasets for larger subpopulations. Specifically, we compared adjusted EHR and NHANES results for those age 18-plus and not pregnant who had healthcare contact in the past 12 months, and we compared adjusted HRS and NHANES results for those age 50-plus regardless of whether they had healthcare contact. The patterns of prevalence and control were similar in those analyses (data not shown).

## Discussion

### Results across data sources

We tested methods for improved diabetes surveillance by exploring three novel data sources for state-level estimation of diabetes comorbidities and ABC control. We did not expect the prevalence and control estimates to align completely, even after adjustment. We cannot reach conclusions about which dataset is most accurate; however, we can describe the patterns across data sources, which illuminate their strengths and weaknesses. While almost every general statement about consistency across datasets has an exception in at least one of the states examined, the two surveys—NHANES and HRS—generally aligned well when using them to make state-adjusted synthetic estimates. The adjusted HRS estimates may be more accurate as the larger HRS sample allowed for the analysis of respondent subsets with closer geographic representation to the target states, especially in California and Florida which, in this analysis, did not involve adjustments to any out-of-state data.

Although one prior study documents good performance of EHR data for estimating diabetes prevalence at the local level [7], we found several differences between the EHR estimates and those derived from the surveys; notably that the EHR-based prevalence estimates were consistently lower for undiagnosed diabetes, significantly so in most states. A1c results were not present for 85% of the EHR population age 50 and older, likely because there was no clinical reason to order the test. Some of the untested individuals likely have undiagnosed diabetes, cases that cannot be identified using EHR data, a conclusion that would hold even if other types of laboratory tests were considered. For diagnosed diabetes, the EHR data produced more variable prevalence estimates than the surveys. Because the EHR data are not population-based and are geographically clustered within each state, it is possible that this variability is more influenced by local patterns in healthcare utilization, diagnostic, or coding practices than the survey data.

EHR-based prevalence estimates for hypertension and high cholesterol within the diabetes population were generally below the estimates from the surveys, except in Massachusetts. The lower EHR comorbidity rates in most states may be due to undercounting of undiagnosed cases, as with diabetes. It is possible that individuals in Massachusetts, a state with near full health insurance coverage and citizens who seek healthcare more often [36], are more likely to have chronic conditions diagnosed. For ABC control, the EHR-based rates were again variable, although there were fewer significant differences than in the prevalence analyses. In all three datasets, rates of A1c control were almost always highest, followed by blood pressure control, and non-HDL-C control.

### Implications for surveillance

EHRs are a promising data source for diabetes surveillance as the datasets are large and created at low cost during the routine delivery of health care. However, any EHR-based data source represents only those who sought care in that provider network. The geographic coverage of the data tends to be clustered in certain localities, and there are limited covariates available for adjustments. Further, clinician decisions to administer the tests needed to assess ABC control, or detect undiagnosed cases of chronic diseases, are non-random. In addition, it is possible that some patients were treated at multiple practices and are double counted. All of these factors introduce the potential for bias in EHR-based surveillance estimates, and are likely responsible for the observed variability in the EHR estimates relative to survey-based estimates. Propensity modeling or other approaches for handling missing data may reduce this bias [4, 37, 38]; however, the optimal adjustment for any particular measure A, B, or C, may not be optimal for the other measures since different subgroups of patients are measured for each indicator. In addition, it is difficult to quantify the uncertainty in EHR-based estimates when the goal is population surveillance.

We found relatively high consistency in the prevalence of diabetes, hypertension, and high cholesterol among those 50 years and older between the NHANES and HRS surveys. This was reassuring since they use similar case ascertainment and data collection methodologies. These surveys did not have state-based samples among our five states for our study years, although NHANES has produced a California file that aggregates and reweights data from four NHANES cycles (2007–2014). We did not analyze this dataset as it did not match the time period of this analysis [39]. The lack of state-based samples necessitated the use of statistical adjustments to create state-level estimates. In previous studies, synthetic adjustment of NHANES data noticeably changed state estimates [15], and improved accuracy relative to a gold standard [4]. A description of alternative methods that might be used can be found in Rao and Molina [40].

## Conclusions

EHR data availability for research and epidemiology, and methods for analyzing it, are likely to improve over time as the use of these systems expands. Inclusion of key covariates such as race would allow for reductions in their biases. There are few sources of EHR data available for research outside of the health systems that generate the data. The DARTNet Institute, which compiles data from numerous providers and makes it available for research, is one notable exception. As the use of EHRs continues to expand, organizations that compile and standardize the data for research will be integral to their use for state-level diabetes surveillance. While our analysis is limited to five states, it suggests areas for future research that could enhance national surveillance efforts and provide state-level estimates of measures obtained primarily in clinical or laboratory settings.

## Supplementary Information

**Additional file 1: Figure A1.** EHR Data File Preparation Workflow. *Required 2 blood pressure readings in range to quality as a case of hypertension

**Additional file 2: Table A1.** Diabetes Case Definition Details for Each Data Source. **Table A2.** Hypertension Case Definition Details for Each Data Source. **Table A3.** High Cholesterol Case Definition Details for Each Data Source.

## Data Availability

NHANES data used in the study are publically available at https://wwwn.cdc.gov/nchs/nhanes/continuousnhanes/default.aspx?BeginYear=2011. HRS and DARTNet EHR data are available from the Health and Retirement Study and DARTNet Institute respectively. Restrictions apply to the use of these data, which were used under license for the current study, and so are not publicly available. Data are, however, available from the authors upon reasonable request and with permission of the licensors.

## Abbreviations

ABC: Hemoglobin A1c
CI: Confidence interval
EHR: Electronic health record
FPG: Fasting plasma glucose
HDL-C: High-density lipoprotein cholesterol
HRS: Health and Retirement Study
LDL-C: Low-density lipoprotein cholesterol
NHANES: National Health and Nutrition Examination Survey
