# Supplementary material for: State-level metabolic comorbidity prevalence and control among adults age 50-plus with diabetes: estimates from electronic health records and survey data in five states
Source: Popul Health Metr. 2022 Dec 2;20:22. doi: 10.1186/s12963-022-00298-z (PMC9719142; doi:10.1186/s12963-022-00298-z)
Supplement: Supplementary file 2 — Additional file 2: Table A1. Diabetes Case Definition Details for Each Data Source. Table A2. Hypertension Case Definition Details for Each Data Source. Table A3. High Cholesterol Case Definition Details for Each Data Source. [file 12963_2022_298_MOESM2_ESM.docx]

**Online Appendix**

Figure A1 shows the EHR data file preparation workflow.

Table A1: Diabetes Case Definition Details for Each Data Source

| Criteria* | Description | Data Source |
| --- | --- | --- |
| Self-report of Diagnosed Diabetes | Answered YES to survey question:  NHANES: “Other than during pregnancy (females only), have you ever been told by a doctor or other health professional that you have diabetes or sugar diabetes?”  HRS: Has a doctor ever told you that you have diabetes or high blood sugar? | NHANES, HRS |
| Diagnoses codes and mapped SNOMED codes for diabetes and related conditions | Diagnosis codes stored as a “condition source value”.   - ICD9 codes for Diabetes Mellitus (DM) with or without complication; Type I or type II: 250.x0 - 250.x3 - ICD9 code for polyneuropathy in diabetes: 357.2 - ICD9 code for diabetic retinopathy: 362.0x - ICD9 code for diabetic cataract: 364.41 - ICD9 code for DM of mother complicating pregnancy, childbirth, or the puerperium unspecified as to episode of care (this is not gestational diabetes): 648.08x - ICD10 DM for above list: over 50 E codes with majority starting with E10 or E11; eleven O24 codes - SNOMED: over 100 codes | EHR |
| Prescription anti-diabetic medication | Members who were prescribed insulin or hypoglycemics/ antihyperglycemics during 2012-2013:   - Alpha-glucosidase inhibitors - Amylin analogs - Insulin - Antidiabetic agent combinations including those with metformin - Meglitinides - Sodium glucose cotransporter 2 (SGLT2) inhibitors - Sulfonylureas or thiazolidinediones | EHR |
| Hemoglobin A1c | If none of the above and Hemoglobin A1c ≥ 6.5, then undiagnosed diabetes = “yes” | NHANES, HRS, EHR |
| Fasting plasma glucose (FPG) | If none of the above and FPG ≥ 126 mg/dl then undiagnosed diabetes = “yes” | NHANES, HRS |

*Individuals who meet the conditions in any row are classified as a case.

Table A2: Hypertension Case Definition Details for Each Data Source

| Criteria* | Description | Data Source |
| --- | --- | --- |
| Diagnoses codes and mapped SNOMED codes for hypertension | Diagnosis codes stored as a “condition source value”.   - ICD9 codes for hypertension: 401.x-405.x - ICD10 DM for above list: I10, I11.x, I12.x, I13.x, I15.x - SNOMED: over 100 codes | EHR |
| Prescription hypertensive medication | The DRUG_CONCEPT_ID in the patient record includes the codes of anti-hypertensive drugs.   - Angiotensin-converting-enzyme (ACE) Inhibitors - Angiotensin II receptor blockers (ARB) - Beta blocking agents - Select calcium channel blockers - Diuretics   Exclude when a solo find of the drug is accompanied by the diagnosis codes for migraines, congestive heart failure, myocardial infarction; diabetes, cardiac arrhythmia, tremor, angina, kidney stones, or portal hypertension. | EHR |
| Self-report of hypertension medication | NHANES: Answered YES to “Are you now taking prescribed medicine because of your high blood pressure/hypertension?  HRS: Answered YES to “In order to lower you blood pressure, are you now taking medication?” | NHANES, HRS |
| Blood pressure readings | Survey site visit: systolic blood pressure > 140 mmHg OR Diastolic blood pressure > 90 mmHg (NHANES and HRS uses the mean of all available readings).  BP readings from DARTNet observation_concept_id data: systolic > 140 mmHg OR  Diastolic blood pressure > 90 mmHg two or more times in the period examined. | NHANES, HRS, EHR |

*Individuals who meet the conditions in any row are classified as a case.

Table A3. High Cholesterol Case Definition Details for Each Data Source

| Criteria* | Description | Data Source |
| --- | --- | --- |
| Diagnoses codes and mapped SNOMED codes for high cholesterol | Diagnosis codes stored as a “condition source value”.   - ICD9 codes for hypercholesterolemia, hyperglyceridemia, mixed hyperlipidemia, hyperchylomicronemia, other and unspecified hyperlipidemia, other lipoprotein deficiencies, and other disorders of lipoid metabolism: 272.x - ICD10 for the above codes: E75.2x, E77.0, E77.1, E78.x, E88.1, E88.89 - SNOMED: over 100 codes | EHR |
| Prescription cholesterol-lowering medication | The following anti-hyperlipidemia agents:   - HMG CoA reductase inhibitors - Fibrates - Bile acid sequestrants - Other lipid modifying agents - HMG CoA reductase inhibitors in combination with other lipid modifying agents | EHR |
| Self-report of cholesterol-lowering medication | NHANES: Answered YES to “Are you now following this advice {from doctor or other health professional} to take prescribed medicine {to lower your blood cholesterol}”?  HRS: Answered YES to “Do you regularly take prescription medications to help lower your cholesterol?” | NHANES, HRS |
| Non-High Density Lipid (HDL) value | Non-HDL was calculated using an individual’s total cholesterol and high-density lipoprotein collected on the same day. [TOTAL-HDL]  Non-HDL ≥ 130 mg/dL = high cholesterol | NHANES, HRS, EHR |

*Individuals who meet the conditions in any row are classified as a case.
