# Supplementary material for: State-level metabolic comorbidity prevalence and control among adults age 50-plus with diabetes: estimates from electronic health records and survey data in five states
Source: Popul Health Metr. 2022 Dec 2;20:22. doi: 10.1186/s12963-022-00298-z (PMC9719142; doi:10.1186/s12963-022-00298-z)
Supplement: Supplementary file 2 [file 12963_2022_298_MOESM1_ESM.docx]

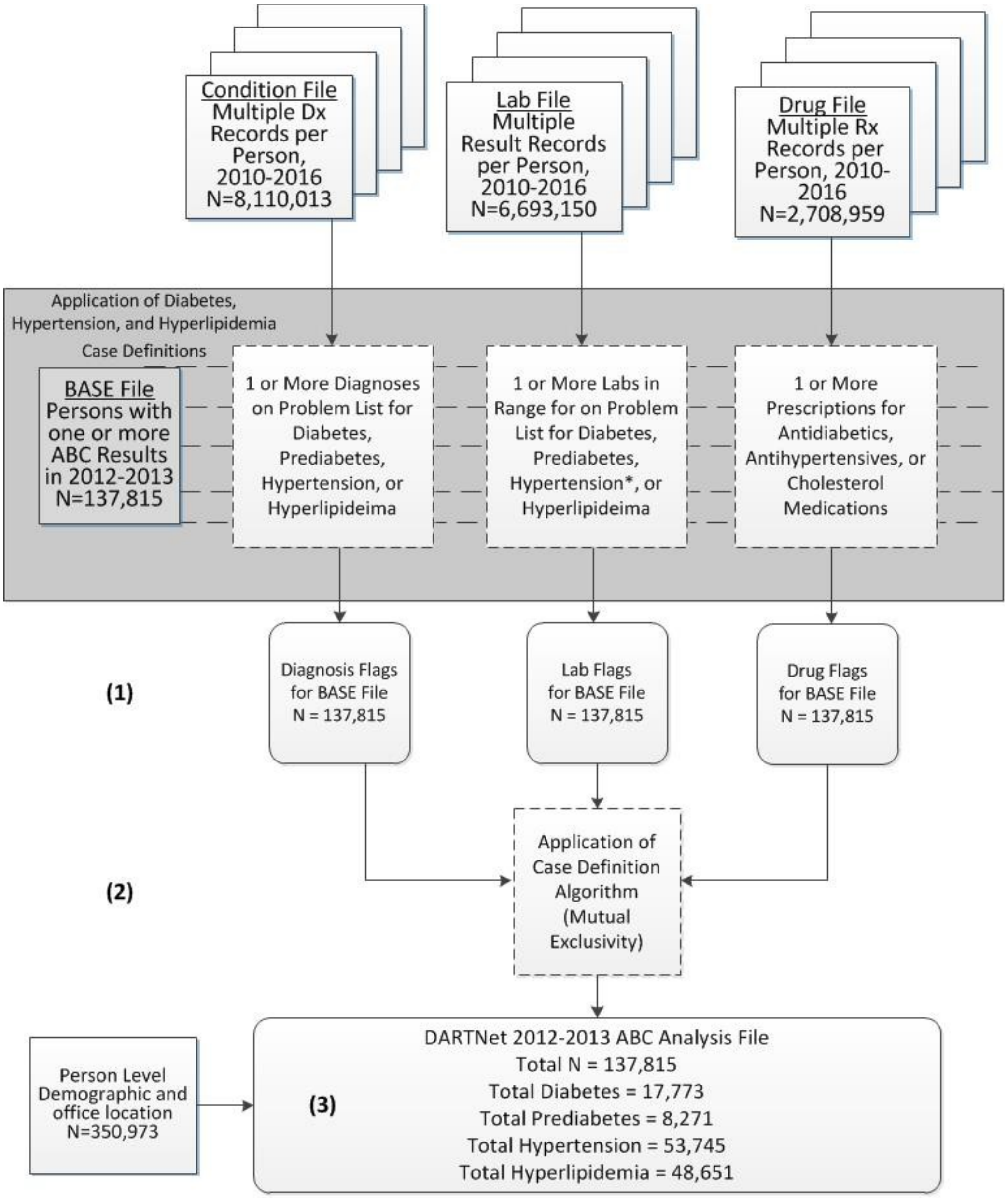
Condition File
Multiple Dx Records per Person, 2010-2016
N=8,110,013
Lab File
Multiple Result Records per Person, 2010-2016
N=6,693,150
Drug File
Multiple Rx Records per Person, 2010-2016
N=2,708,959
Application of Diabetes, Hypertension, and Hyperlipidemia Case Definitions
BASE File
Persons with one or more ABC Results in 2012-2013
N=137,815
1 or More Diagnoses on Problem List for Diabetes, Prediabetes, Hypertension, or Hyperlipideima
1 or More Labs in Range for on Problem List for Diabetes, Prediabetes, Hypertension*, or Hyperlipideima
1 or More Prescriptions for Antidiabetics, Antihypertensives, or Cholesterol Medications
(1)
Diagnosis Flags for BASE File
N = 137,815
Lab Flags for BASE File
N = 137,815
Drug Flags for BASE File
N = 137,815
(2)
Application of Case Definition Algorithm (Mutual Exclusivity)
Person Level Demographic and office location
N=350,973
(3)
DARTNet 2012-2013 ABC Analysis File
Total N = 137,815
Total Diabetes = 17,773
Total Prediabetes = 8,271
Total Hypertension = 53,745
Total Hyperlipidemia = 48,651
